# Crystal structure of bis­(prop-2-yn-1-yl) 5-nitro­isophthalate

**DOI:** 10.1107/S2056989015009846

**Published:** 2015-05-30

**Authors:** K. S. Ezhilarasi, Sivasamy Selvarani, Perumal Rajakumar, B. K. Revathi, G. Usha

**Affiliations:** a PG and Research Department of Physics, Queen Mary’s College, Chennai-4, Tamilnadu, India; b Department of Organic Chemistry, University of Madras, Guindy Campus, Chennai-25, India

**Keywords:** crystal structure, 5-nitro­isophthalate, prop-2-yn-1-yl, twofold rotation symmetry, C—H⋯O hydrogen bonding

## Abstract

The whole mol­ecule of the title compound, C_14_H_9_NO_6_, is generated by twofold rotation symmetry; the twofold axis bis­ects the nitro group and the benzene ring. The nitro group is inclined to the benzene ring by 14.42 (9)°. The prop-2-yn-1-yl groups are inclined to the benzene ring by 13 (2)° and to each other by 24 (3)°; one directed above the plane of the benzene ring and the other below. In the crystal, mol­ecules are linked *via* pairs of C—H⋯O hydrogen bonds, forming inversion dimers with an *R*_2_^2^(18) ring motif. The dimers are linked by further C—H⋯O hydrogen bonds, forming sheets lying parallel to (100).

## Related literature   

For the biological activities of carboxyl­ates, see: Choudhary *et al.* (2002 ▸). For the uses and properties of nitro­aromatics, see: Lee *et al.* (2013 ▸); Somerville *et al.* (1995 ▸).
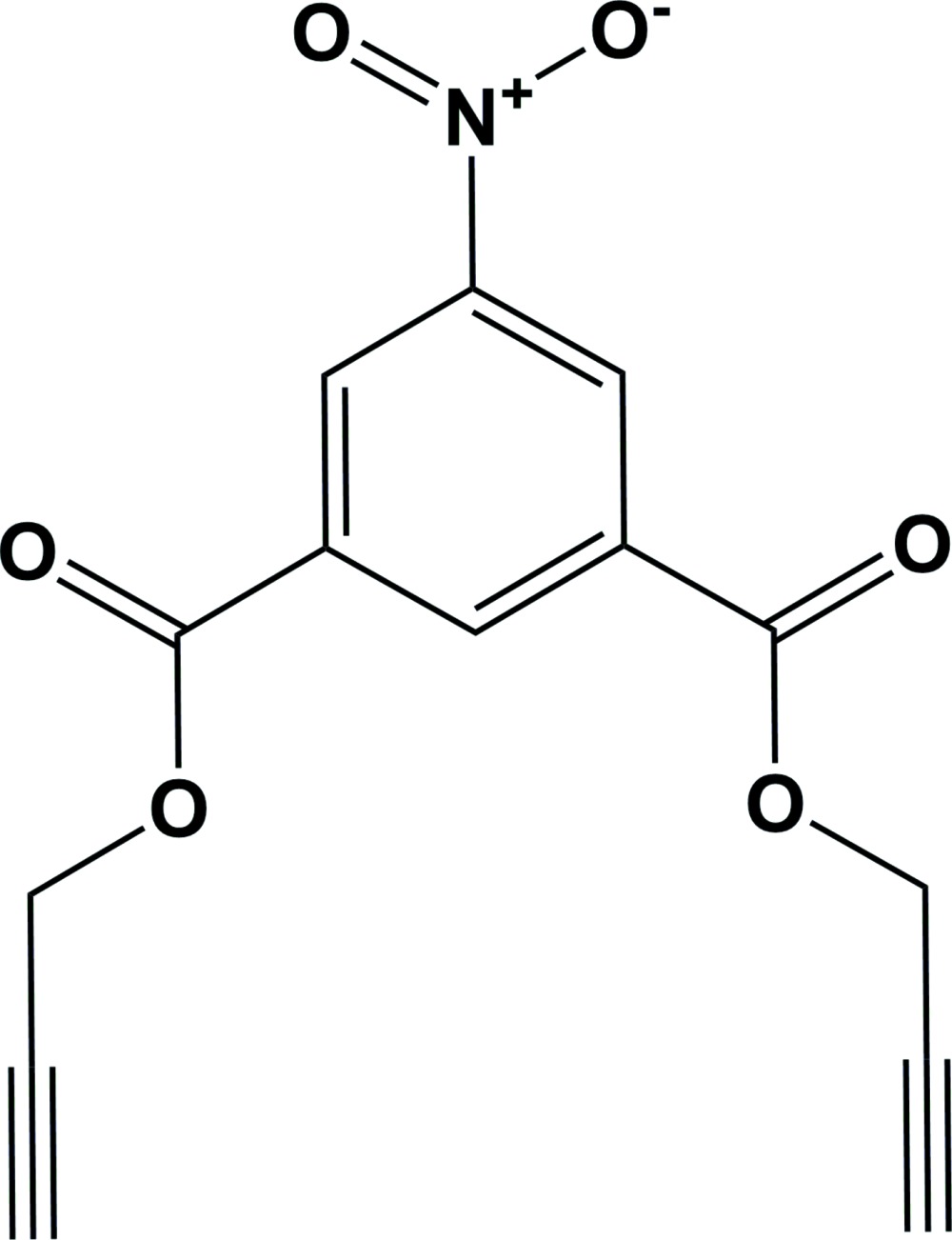


## Experimental   

### Crystal data  

C_14_H_9_NO_6_
*M* *_r_* = 287.22
Orthorhombic, 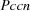

*a* = 6.679 (5) Å
*b* = 11.679 (5) Å
*c* = 16.503 (5) Å
*V* = 1287.3 (12) Å^3^
*Z* = 4
Mo *K*α radiation
μ = 0.12 mm^−1^
*T* = 293 K
0.30 × 0.25 × 0.20 mm

### Data collection  

Bruker Kappa APEXII CCD diffractometer
Absorption correction: multi-scan (*SADABS*; Bruker, 2008 ▸) *T*_min_ = 0.965, *T*_max_ = 0.977
6369 measured reflections
1613 independent reflections
1316 reflections with *I* > 2σ(*I*)
*R*_int_ = 0.021

### Refinement  

*R*[*F* ^2^ > 2σ(*F* ^2^)] = 0.038
*wR*(*F* ^2^) = 0.136
*S* = 0.73
1523 reflections
98 parameters
H-atom parameters constrained
Δρ_max_ = 0.26 e Å^−3^
Δρ_min_ = −0.21 e Å^−3^

### 

Data collection: *APEX2* (Bruker, 2008 ▸); cell refinement: *SAINT* (Bruker, 2008 ▸); data reduction: *SAINT*; program(s) used to solve structure: *SHELXS97* (Sheldrick, 2008 ▸); program(s) used to refine structure: *SHELXL97* (Sheldrick, 2008 ▸); molecular graphics: *ORTEP-3 for Windows* (Farrugia, 2012 ▸); software used to prepare material for publication: *SHELXL97* and *PLATON* (Spek, 2009 ▸).

## Supplementary Material

Crystal structure: contains datablock(s) I, Global. DOI: 10.1107/S2056989015009846/su5137sup1.cif

Structure factors: contains datablock(s) I. DOI: 10.1107/S2056989015009846/su5137Isup2.hkl

Supporting information file. DOI: 10.1107/S2056989015009846/su5137Isup3.cml

**Fig. 1.**
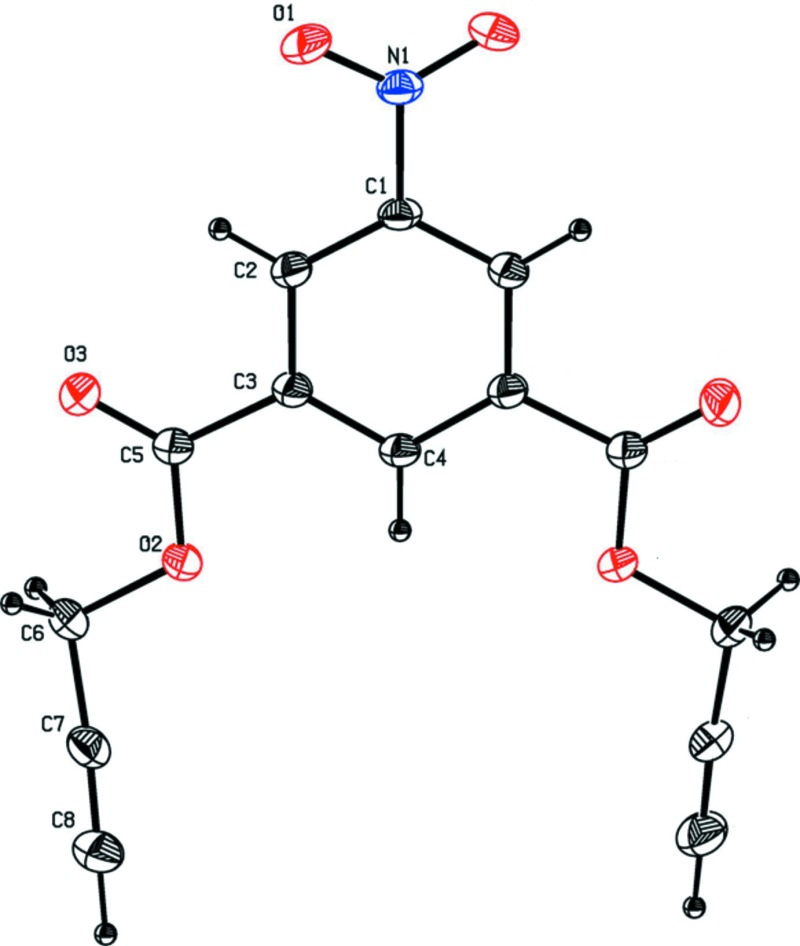
The molecular structure of the title compound, with atom labelling. Displacement ellipsoids are drawn at the 30% probability level. The unlabelled atoms are related to the labelled atoms by twofold rotation symmetry [symmetry code: (i) -x + 3/2, -y + 1/2, z].

**Fig. 2.**
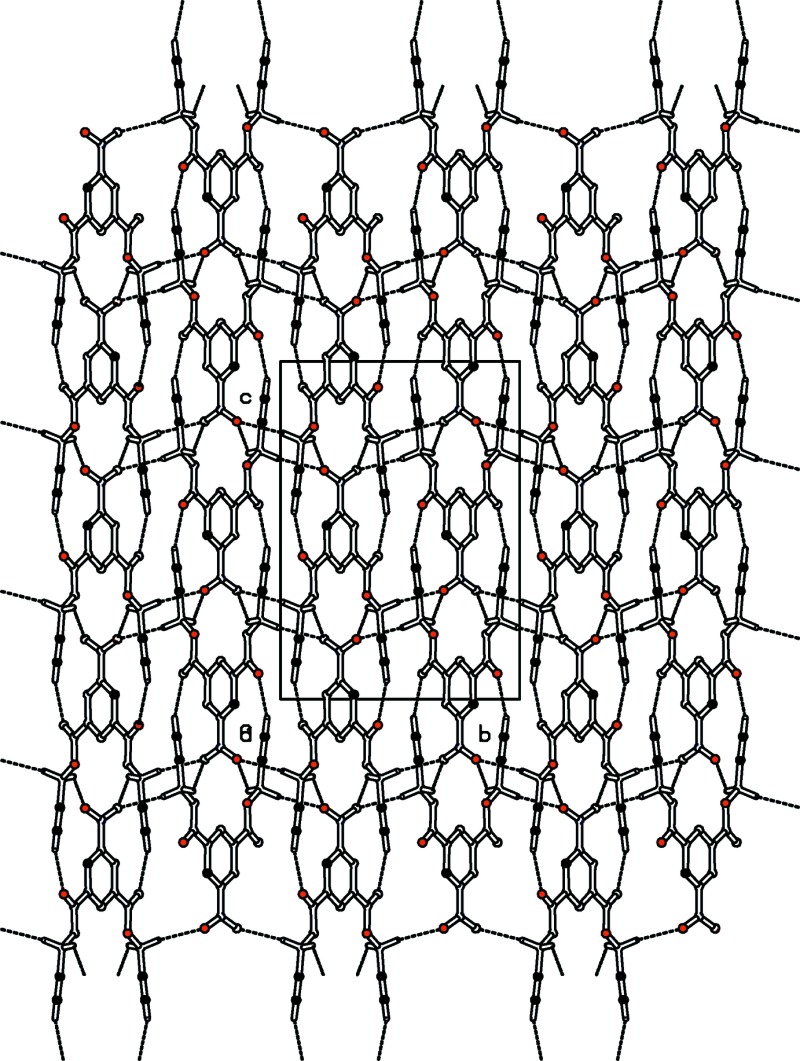
A view along the a axis of the crystal packing of the title compound. The dashed lines indicate hydrogen bonds (see Table 1 for details).

Click here for additional data file.x y z . DOI: 10.1107/S2056989015009846/su5137fig1.tif
The mol­ecular structure of the title compound, with atom labelling. Displacement ellipsoids are drawn at the 30% probability level. The unlabelled atoms are related to the labelled atoms by twofold rotation symmetry [symmetry code: (i) −*x* + 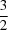, −*y* + 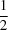, *z*].

a . DOI: 10.1107/S2056989015009846/su5137fig2.tif
A view along the *a* axis of the crystal packing of the title compound. The dashed lines indicate hydrogen bonds (see Table 1 for details).

CCDC reference: 1402145

Additional supporting information:  crystallographic information; 3D view; checkCIF report

## Figures and Tables

**Table 1:** Hydrogen-bond geometry (, )

*D*H*A*	*D*H	H*A*	*D* *A*	*D*H*A*
C6H6*A*O1^i^	0.97	2.46	3.334(2)	150
C6H6*B*O1^ii^	0.97	2.57	3.313(2)	134
C8H8O3^ii^	0.93	2.50	3.251(2)	138

Symmetry codes: (i) 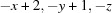; (ii) 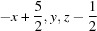.

## supplementary crystallographic information

### S1. Comments

Carboxyl­ates have promising activity against various anti­tumor cells (Choudharyl *et al.*, 2002). Nitro­aromatic compounds are used in the production of dyes, plastics, high explosives, pharmaceuticals, and pesticides (Somerville *et al.*, 1995). Nitro­benzene is mostly used in the synthesis of aniline and in the production of benzidine, quinolone and azo­benzene (Lee *et al.*, 2013).

In the title compound, Fig. 1, the two-fold rotation bis­ects the benzene ring and the nitro group; atoms C1, C4, H4 and N1 lie on the two-fold rotation axis. The nitro group is inclined to the benzene ring by 14.42 (9) °. The prop-2-yn-1-yl groups are inclined to the benzene ring by 13 (2) ° and to each other by 24 (3) °; one directed above the plane of the benzene ring and the other below.

In the crystal, molecules are linked *via* pairs of C—H···O hydrogen bonds forming inversion dimers with an *R*_2_^2^(18) ring motif (Table 1). The dimers are linked by further C—H···O hydrogen bonds forming sheets lying parallel to (100); see Table 1 and Fig. 2.

### S2. Synthesis and crystallization

The title compound was synthesized by Steglich esterification of 5-nitro isophthalic acid (1 equiv) which together with propargyl alcohol (2.2 equiv) was added at 273 K to DMAP (2.5 equiv) and DCC (2.2 equiv) in di­chloro­methane (100 ml). The mixture was stirred under nitro­gen at room temperature for 24 h. The white precipitate that formed was filtered off and washed with DCM (150 ml) and brine (150 ml), then dried over Na_2_SO_4_, filtered and evaporated to afforded the title compound. It was purified by column chromatography using CHCl_3_:hexane (9:1) as a eluent. Crystals were obtained by slow evaporation of the solvent.

### S3. Refinement

Crystal data, data collection and structure refinement details are summarized in Table 2. H atoms were positioned geometrically and treated as riding atoms: C—H = 0.93-0.97 Å with U_iso_(H) = 1.5U_eq_(C) for methyl H atoms and 1.2U_eq_(C) for other H atoms.

### Crystal data

**Table:**

C_14_H_9_NO_6_	*F*(000) = 592
*M**_r_* = 287.22	*D*_x_ = 1.482 Mg m^−^^3^
Orthorhombic, *P**c**c**n*	Mo *K*α radiation, λ = 0.71073 Å
Hall symbol: -P 2ab 2ac	θ = 2.5–28.4°
*a* = 6.679 (5) Å	µ = 0.12 mm^−^^1^
*b* = 11.679 (5) Å	*T* = 293 K
*c* = 16.503 (5) Å	Block, colourless
*V* = 1287.3 (12) Å^3^	0.30 × 0.25 × 0.20 mm
*Z* = 4	

### Data collection

**Table:**

Bruker Kappa APEXII CCD diffractometer	1613 independent reflections
Radiation source: fine-focus sealed tube	1316 reflections with *I* > 2σ(*I*)
Graphite monochromator	*R*_int_ = 0.021
ω and φ scan	θ_max_ = 28.4°, θ_min_ = 2.5°
Absorption correction: multi-scan (*SADABS*; Bruker, 2008)	*h* = −8→8
*T*_min_ = 0.965, *T*_max_ = 0.977	*k* = −15→7
6369 measured reflections	*l* = −12→22

### Refinement

**Table:**

Refinement on *F*^2^	Secondary atom site location: difference Fourier map
Least-squares matrix: full	Hydrogen site location: inferred from neighbouring sites
*R*[*F*^2^ > 2σ(*F*^2^)] = 0.038	H-atom parameters constrained
*wR*(*F*^2^) = 0.136	*w* = 1/[σ^2^(*F*_o_^2^) + (0.1246*P*)^2^ + 0.5331*P*] where *P* = (*F*_o_^2^ + 2*F*_c_^2^)/3
*S* = 0.73	(Δ/σ)_max_ < 0.001
1523 reflections	Δρ_max_ = 0.26 e Å^−^^3^
98 parameters	Δρ_min_ = −0.21 e Å^−^^3^
0 restraints	Extinction correction: *SHELXL97* (Sheldrick, 2008), Fc^*^=kFc[1+0.001xFc^2^λ^3^/sin(2θ)]^-1/4^
Primary atom site location: structure-invariant direct methods	Extinction coefficient: 0.030 (5)

### Special details

**Table:**

Geometry. All e.s.d.'s (except the e.s.d. in the dihedral angle between two l.s. planes) are estimated using the full covariance matrix. The cell e.s.d.'s are taken into account individually in the estimation of e.s.d.'s in distances, angles and torsion angles; correlations between e.s.d.'s in cell parameters are only used when they are defined by crystal symmetry. An approximate (isotropic) treatment of cell e.s.d.'s is used for estimating e.s.d.'s involving l.s. planes.
Refinement. Refinement of *F*^2^ against ALL reflections. The weighted *R*-factor *wR* and goodness of fit *S* are based on *F*^2^, conventional *R*-factors *R* are based on *F*, with *F* set to zero for negative *F*^2^. The threshold expression of *F*^2^ > σ(*F*^2^) is used only for calculating *R*-factors(gt) *etc*. and is not relevant to the choice of reflections for refinement. *R*-factors based on *F*^2^ are statistically about twice as large as those based on *F*, and *R*- factors based on ALL data will be even larger.

### Fractional atomic coordinates and isotropic or equivalent isotropic displacement parameters (Å^2^)

**Table:**

	*x*	*y*	*z*	*U*_iso_*/*U*_eq_	
C1	0.7500	0.2500	0.05352 (9)	0.0336 (4)	
C2	0.90289 (17)	0.30592 (10)	0.01386 (7)	0.0351 (3)	
H2	1.0033	0.3433	0.0426	0.042*	
C3	0.90264 (16)	0.30486 (10)	−0.07063 (7)	0.0329 (3)	
C4	0.7500	0.2500	−0.11315 (9)	0.0328 (4)	
H4	0.7500	0.2500	−0.1695	0.039*	
C5	1.07371 (18)	0.36348 (11)	−0.11169 (7)	0.0377 (3)	
C6	1.2259 (2)	0.41344 (12)	−0.23491 (7)	0.0431 (3)	
H6A	1.2527	0.4888	−0.2127	0.052*	
H6B	1.3450	0.3669	−0.2284	0.052*	
C7	1.17465 (19)	0.42270 (11)	−0.32014 (8)	0.0412 (3)	
C8	1.1422 (2)	0.43439 (16)	−0.38961 (9)	0.0565 (4)	
H8	1.1166	0.4436	−0.4446	0.068*	
N1	0.7500	0.2500	0.14284 (8)	0.0377 (4)	
O1	0.86053 (16)	0.31731 (9)	0.17767 (6)	0.0525 (3)	
O3	1.21118 (18)	0.40639 (12)	−0.07644 (6)	0.0700 (4)	
O2	1.05835 (13)	0.36103 (8)	−0.19240 (5)	0.0404 (3)	

### Atomic displacement parameters (Å^2^)

**Table:**

	*U*^11^	*U*^22^	*U*^33^	*U*^12^	*U*^13^	*U*^23^
C1	0.0357 (8)	0.0445 (8)	0.0205 (8)	0.0030 (6)	0.000	0.000
C2	0.0344 (6)	0.0445 (6)	0.0263 (6)	−0.0024 (4)	−0.0023 (4)	−0.0013 (4)
C3	0.0328 (6)	0.0407 (6)	0.0254 (6)	−0.0011 (4)	0.0013 (4)	0.0007 (4)
C4	0.0355 (8)	0.0411 (8)	0.0218 (7)	−0.0010 (6)	0.000	0.000
C5	0.0378 (6)	0.0483 (7)	0.0270 (6)	−0.0062 (5)	0.0000 (5)	0.0015 (5)
C6	0.0392 (7)	0.0559 (7)	0.0342 (7)	−0.0110 (5)	0.0065 (5)	0.0047 (5)
C7	0.0401 (6)	0.0463 (6)	0.0370 (7)	−0.0021 (5)	0.0090 (5)	0.0045 (5)
C8	0.0549 (8)	0.0771 (10)	0.0376 (7)	−0.0064 (7)	0.0045 (6)	0.0094 (7)
N1	0.0369 (7)	0.0529 (8)	0.0234 (7)	0.0042 (6)	0.000	0.000
O1	0.0568 (6)	0.0728 (7)	0.0278 (5)	−0.0089 (5)	−0.0065 (4)	−0.0078 (4)
O3	0.0606 (7)	0.1147 (11)	0.0345 (5)	−0.0461 (7)	−0.0069 (5)	0.0068 (6)
O2	0.0393 (5)	0.0563 (6)	0.0256 (5)	−0.0124 (4)	0.0048 (3)	−0.0012 (3)

### Geometric parameters (Å, º)

**Table:**

C1—C2^i^	1.3775 (15)	C5—O2	1.3363 (15)
C1—C2	1.3775 (15)	C6—C7	1.4517 (18)
C1—N1	1.474 (2)	C6—O2	1.4555 (15)
C2—C3	1.3944 (16)	C6—H6A	0.9700
C2—H2	0.9300	C6—H6B	0.9700
C3—C4	1.3937 (15)	C7—C8	1.175 (2)
C3—C5	1.4944 (17)	C8—H8	0.9300
C4—C3^i^	1.3937 (15)	N1—O1	1.2220 (12)
C4—H4	0.9300	N1—O1^i^	1.2220 (12)
C5—O3	1.1970 (17)		
			
C2^i^—C1—C2	123.27 (14)	O2—C5—C3	112.55 (10)
C2^i^—C1—N1	118.36 (7)	C7—C6—O2	108.50 (11)
C2—C1—N1	118.36 (7)	C7—C6—H6A	110.0
C1—C2—C3	118.03 (11)	O2—C6—H6A	110.0
C1—C2—H2	121.0	C7—C6—H6B	110.0
C3—C2—H2	121.0	O2—C6—H6B	110.0
C4—C3—C2	120.56 (11)	H6A—C6—H6B	108.4
C4—C3—C5	122.79 (11)	C8—C7—C6	176.19 (14)
C2—C3—C5	116.64 (10)	C7—C8—H8	180.0
C3—C4—C3^i^	119.53 (14)	O1—N1—O1^i^	123.89 (15)
C3—C4—H4	120.2	O1—N1—C1	118.06 (7)
C3^i^—C4—H4	120.2	O1^i^—N1—C1	118.06 (7)
O3—C5—O2	123.52 (11)	C5—O2—C6	114.36 (9)
O3—C5—C3	123.92 (12)		
			
C2^i^—C1—C2—C3	−0.45 (8)	C2—C3—C5—O2	−178.24 (10)
N1—C1—C2—C3	179.55 (8)	O2—C6—C7—C8	166 (2)
C1—C2—C3—C4	0.91 (15)	C2^i^—C1—N1—O1	−165.80 (8)
C1—C2—C3—C5	−178.32 (9)	C2—C1—N1—O1	14.20 (8)
C2—C3—C4—C3^i^	−0.46 (8)	C2^i^—C1—N1—O1^i^	14.20 (8)
C5—C3—C4—C3^i^	178.71 (12)	C2—C1—N1—O1^i^	−165.80 (8)
C4—C3—C5—O3	−176.32 (13)	O3—C5—O2—C6	1.09 (19)
C2—C3—C5—O3	2.9 (2)	C3—C5—O2—C6	−177.80 (10)
C4—C3—C5—O2	2.56 (15)	C7—C6—O2—C5	−170.29 (11)

Symmetry code: (i) −*x*+3/2, −*y*+1/2, *z*.

### Hydrogen-bond geometry (Å, º)

**Table:**

*D*—H···*A*	*D*—H	H···*A*	*D*···*A*	*D*—H···*A*
C6—H6*A*···O1^ii^	0.97	2.46	3.334 (2)	150
C6—H6*B*···O1^iii^	0.97	2.57	3.313 (2)	134
C8—H8···O3^iii^	0.93	2.50	3.251 (2)	138

Symmetry codes: (ii) −*x*+2, −*y*+1, −*z*; (iii) −*x*+5/2, *y*, *z*−1/2.
