# Supplementary figures and images for: Crystal structure of bis­(prop-2-yn-1-yl) 5-nitro­isophthalate
Source: Acta Crystallogr E Crystallogr Commun. 2015 May 30;71(Pt 6):o435. doi: 10.1107/S2056989015009846 (PMC4459362; doi:10.1107/S2056989015009846)

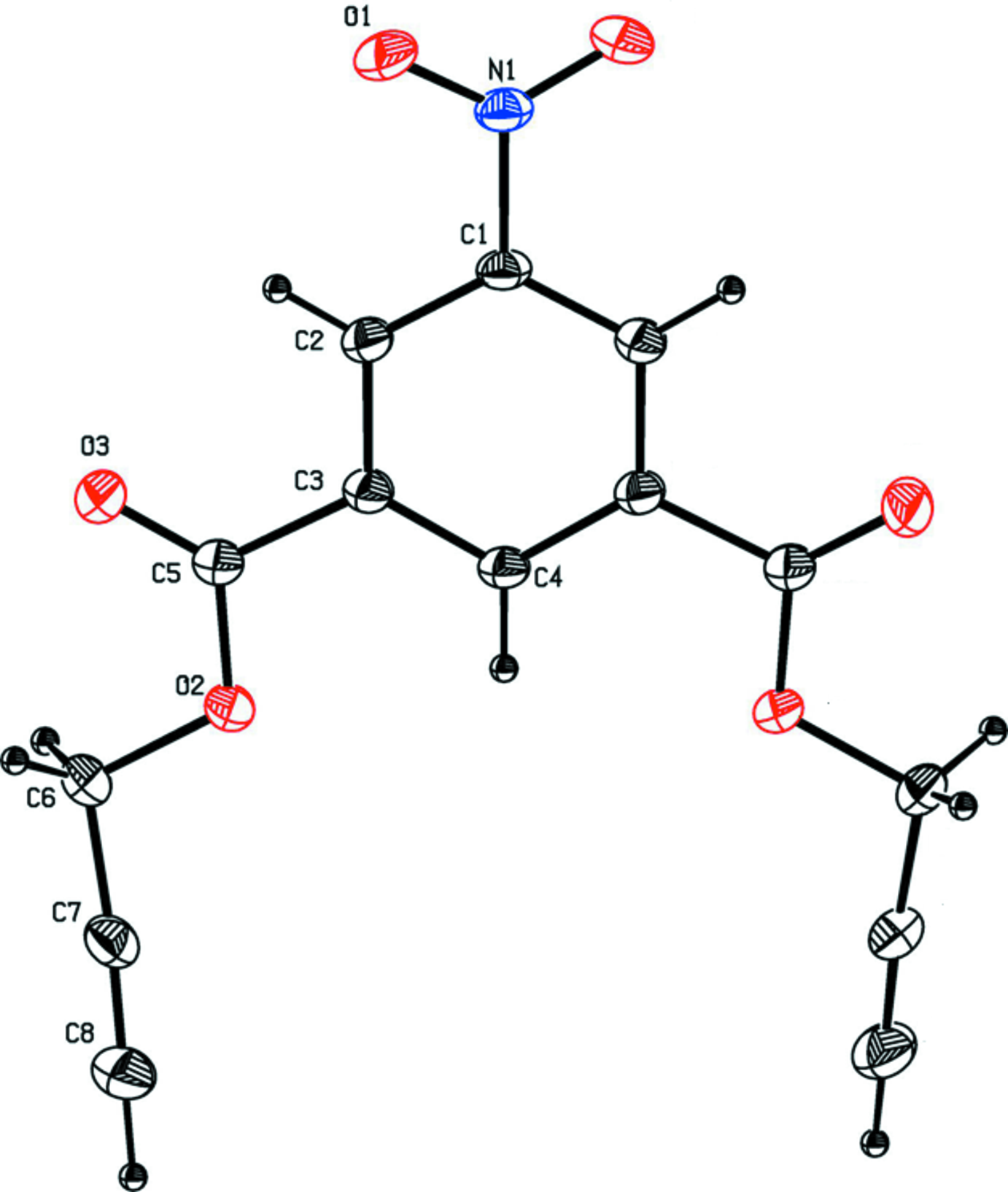

Supplement: Supplementary file 4 [file e-71-0o435-fig1.tif]

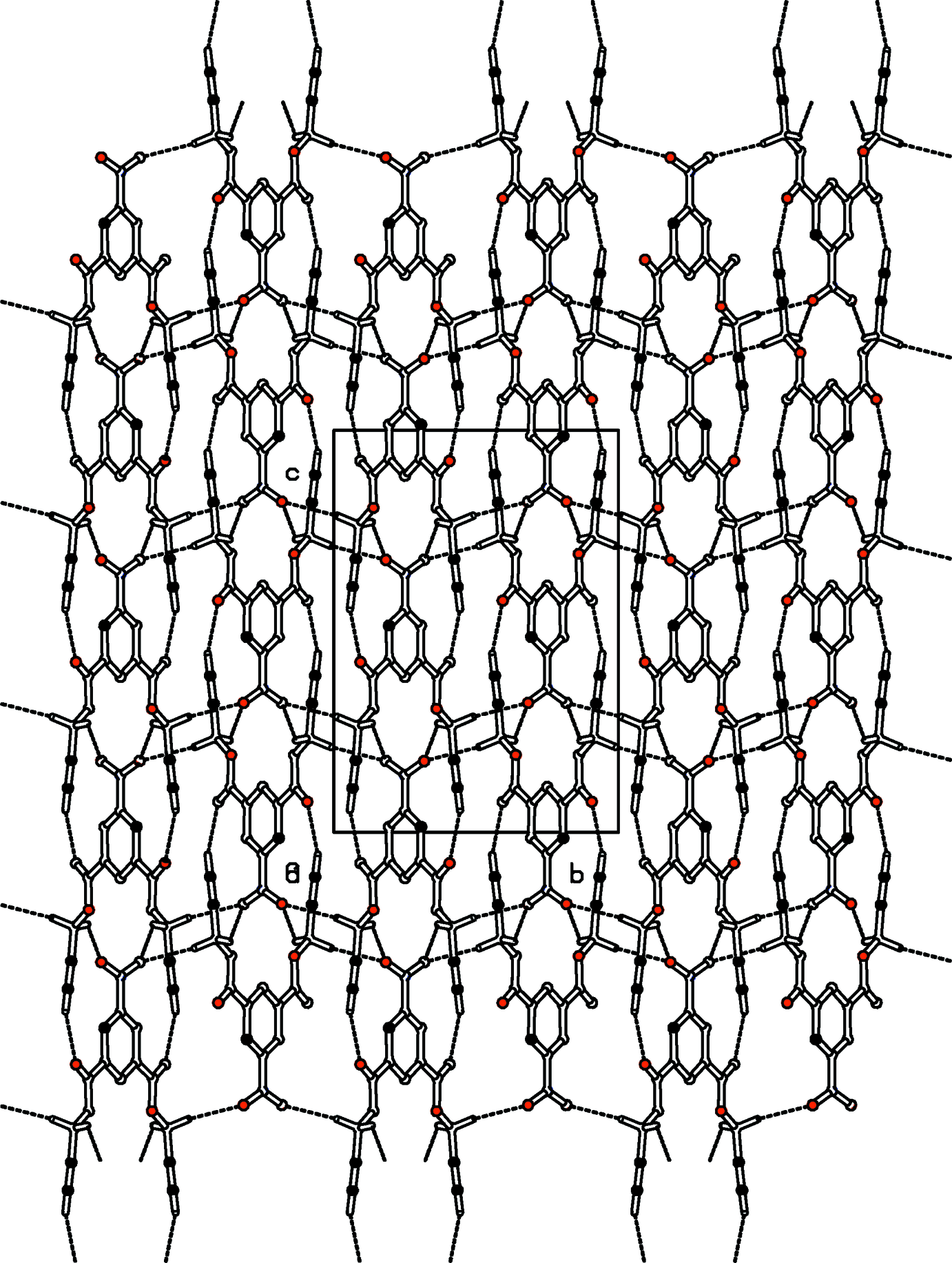

Supplement: Supplementary file 5 [file e-71-0o435-fig2.tif]
